# The association between compliance and persistence with bisphosphonate therapy and fracture risk: A review

**DOI:** 10.1186/1471-2474-8-97

**Published:** 2007-09-26

**Authors:** Jonathan Adachi, Niall Lynch, Hans Middelhoven, Manjit Hunjan, Warren Cowell

**Affiliations:** 1 McMaster University, Hamilton, Canada; 2 Global Health Outcomes, GlaxoSmithKline, Greenford, UK; 3 Economic Value Strategy, F. Hoffmann-La Roche AG, Basle, Switzerland; 4 Health Outcomes Research, GlaxoSmithKline UK Ltd, Stockley Park, UK; 5 Healthcare Management, Roche Products Limited, Welwyn Garden City, UK

## Abstract

**Background:**

Sub optimal levels of compliance and persistence with bisphosphonates are potentially compromising the reduction of post menopausal osteoporotic (PMO) fracture risk.

**Methods:**

A structured literature search (1990–2006) was performed to identify primary research studies evaluating the relationship between compliance and persistence with bisphosphonates and post menopausal osteoporotic (PMO) fracture risk in clinical practice. Search criteria were: bisphosphonates; osteoporosis/osteopenia in postmenopausal women; all types of fractures; compliance and persistence.

**Results:**

Only two retrospective studies using prescription databases have specifically evaluated bisphosphonates.

A cohort study tracking 35,537 women reported that in those with a Medication Possession Ratio (MPR) of ≥80% over 24 months the risk of fracture was lower than in those with an MPR of <80% (8.5% v 10.7%, p < 0.001, Relative Risk Reduction (RRR) 21%). In women who persisted with treatment (refill gap <30 days) the risk of fracture was also lower (7.7% v 10.3%, p < 0.001, RRR 29%).

A nested case control study reported that 12 months persistence (refill gap <50% previous prescription (Rx) length) was associated with a 26% reduced risk of fracture (p < 0.05) and 24 months with a 32% reduced risk (p < 0.05).

Four other studies, not specific to bisphosphonates, reported that compliance ≥12 months decreased fracture risk by ~25%.

**Conclusion:**

Sub optimal compliance and persistence with bisphosphonates is not providing the best possible protection against the risk of PMO fracture, however, more research is needed to delineate this relationship in clinical practice.

## Background

Osteoporosis is characterised by low bone density, deterioration of bone tissue and increased susceptibility to fragility fractures [1] most commonly involving the hip, vertebrae and distal radius [2].

International treatment guidelines recommend the use of bisphosphonates as first line therapy, with the prime objective of reducing the number of osteoporotic fractures [3-6]. This class of products is now firmly established as first line therapy with clinical trials having demonstrated that treatment significantly reduces the incidence of both vertebral and non vertebral fractures [4]. In placebo controlled trials of alendronate and risedronate the relative risk reduction (RRR) for vertebral fractures has been reported as 50–60%, for hip fractures as 44–60% [4] and for non vertebral fractures as 51% [7]. For ibandronate users the risk of new vertebral fractures was reduced by up to 62% (p = 0.0001) [8].

The effectiveness of treatments for chronic conditions is generally compromised by sub optimal compliance and persistence with treatment, particularly in asymptomatic diseases [9-11] and a number of studies [12-15] have reported poor levels of compliance and persistence with commonly used drug therapies for osteoporosis including bisphosphonates [16]. Approximately half of all patients do not take bisphosphonates regularly [17] or continue with treatment [18] for at least 12 months, with many discontinuing soon after initiation [19]. While less frequent dosing regimens have significantly improved compliance and persistence, adherence to treatment remains sub optimal [20,21] and is likely to be compromising the benefits of reduced fracture risk demonstrated in clinical trials.

The aim of this study was to identify primary research studies evaluating the association between compliance and persistence with bisphosphonates and the risk of osteoporotic fracture in clinical practice, focusing on postmenopausal women (>45 years) with osteoporosis or osteopenia.

## Methods

A structured search of bibliographic sources, including MEDLINE^®^; EMBASE^® ^(January 1990 – September 2006); The Cochrane Library Databases (Issue 3, 2006, The Cochrane Database of Systematic Reviews (CDSR), the Database of Abstracts of Reviews of Effects (DARE), the Central Health Technology Assessment (HTA) database; and the NHS Economic Evaluation database (HEED), was performed. The search was intended to identify primary research studies evaluating the relationship between compliance and persistence with bisphosphonates and post menopausal osteoporotic fracture risk in clinical practice. The search strategy devised in conjunction with the Royal Society of Medicine Library in London was based on four main criteria:

- Bisphosphonates as a broad term, as well as specific bisphosphonates (e.g. ibandronate, alendronate, risedronate, etidronate, clondronate, pamidronate, zoledronate etc);

- All types of osteoporosis including osteopenia linked to postmenopausal women. (Although all of the databases searched had an indexing term to specifically describe postmenopausal osteoporosis, for completeness, the broader term for osteoporosis was also searched and combined with terms to describe post menopausal and subjects aged over 45 years);

- All types of fractures including hip, wrist, vertebral and non vertebral;

- Compliance, non compliance, persistence, non persistence, adherence and treatment refusal.

Indexing terms represented in the descriptor fields of references and free-text terms appearing in the title or abstract were included in the search. Free-text terms were also searched for in the full text of Cochrane Reviews in the CDSR. All study types were included (e.g. randomised controlled trials, observational studies, etc.). The search was restricted to female human subjects but papers not gender specific were retained for review. Publications reporting on the use of bisphosphonates for treating pain as a result of bone metastases were excluded. The following publication types were also excluded where possible: letters, editorials, news items, case reports, historical articles and comments. EMBASE^® ^has a publication type "note" and, as it was not clear how this is used in the database, these were also included for review. A search of the bibliographies of identified review articles and those reporting on economic evaluations of bisphosphonates was also made.

To understand the extent and direction of on-going research, the structured literature review was supplemented by manual searching of journal supplements reporting on relevant conference proceedings. A search engine was also used to identify abstracts or posters from conferences which had been posted on the internet by 31st October 2006.

All titles and abstracts retrieved were examined by two reviewers to identify potentially relevant studies and copies of the full text were obtained. Relevant information was summarised into a predefined grid: study reference, country in which study performed, data source used, study period, study population, interventions examined, duration of follow-up, outcome(s) measured as well as a summary of key results and then evaluated.

In the studies reviewed, compliance had been measured and reported as a Medication Possession Ratio (MPR %), defined as the proportion of days within the follow-up period for which patients had prescription cover. Persistence had been measured as the time from initiation to discontinuation of treatment and reported as the number of days from the index prescription to the end of the last prescription issued in the follow-up period.

## Results

After removal of duplicates, a total of 241 potentially relevant references was identified from the searches; 193 from the combined search of MEDLINE^® ^and EMBASE^® ^and 48 from the Cochrane Library databases (15 from CDSR, 22 from CENTRAL and 11 from NHS HEED).

A review of all abstracts identified that the majority of publications (235) had reported mainly on clinical trials evaluating efficacy and safety of different bisphosphonates, observational studies separately examining fracture risk, compliance and persistence, as well as health economic evaluations of bisphosphonates. Only 6 studies had specifically examined the association of compliance and persistence with treatment on fracture rates.

Four studies were non specific to bisphosphonates [13,18,22,23] and it was not possible to examine fracture risk specifically relating to bisphosphonates. Women with a diagnosis of osteoporosis and a prescription for either etidronate, conjugated estrogens or alendronate had been selected from a Canadian Health Insurance Database. The minimum age in this cohort was 45 years and the mean age 68 years. After adjustment for multiple covariates, the RRR in those with an MPR ≥ 80% versus those with an MPR <80% was 16% [13]. In women with osteoporosis who regularly took hormone replacement therapy (HRT) bisphosphonates or raloxifene [18] the risk of hip (OR = 0.38, p < 0.01) and vertebral fractures (OR = 0.60, p < 0.05) was significantly reduced. In those taking any type of medication for osteoporosis, an MPR of <80% was associated with an increased risk of fracture (17% 95%CI 9–25%) [22]. In another database study, a nested case control design was used to compare the level of compliance with medication in women ≥ 45 years who had experienced a fracture compared to those who had not. There was an overall lower risk of fracture in those with an MPR of ≥ 90% compared to those with an MPR of <30% (OR = 0.70, 95%CI 0.52–0.93) [23].

Only two bisphosphonate specific studies [24,25] evaluating daily and weekly bisphosphonate regimens have been reported. The first, a cohort study in the USA [24], examined the risk of total, vertebral, non vertebral and osteoporotic hip fracture in 35,537 bisphosphonate naïve women aged >45 years with a diagnosis of osteoporosis and who had been prescribed daily or weekly regimens of risedronate or alendronate.

In those with an MPR of ≥80% over 24 months (43%), the relative risk of any fracture was 21% lower (p < 0.001) compared to those with MPR <80%. The greatest risk reduction was for hip and vertebral fractures (37%, p < 0.001). In women who persisted with treatment for 24 months (20%) the relative risk of any fracture was 29% lower (p < 0.001) compared to those who did not persist with treatment. Again the most significant decrease in risk was for hip and vertebral fractures (44.5% and 40% respectively, p < 0.001). The relationship between the full range of MPR values (0–1.0) and the probability of fracture indicated that the risk of fracture remained largely unchanged for MPR values of up to approximately 50%, declining with a shallow slope for MPR values between 50% and 75% and then more sharply between 75–100%.

In the subset of women with a diagnosis of post menopausal osteoporosis (n = 6,391), the proportion who were compliant (43%) or persistent (23%) was very similar to that in the overall cohort. Compliance and persistence with therapy was associated with a decrease in the risk of all fractures (22% and 29% respectively, p < 0.001) as well as in hip and vertebral fractures (46.6% and 35.5% respectively, p < 0.001). Covariates that had been adjusted for in these analyses were age, previous fracture history, baseline estrogen and glucocorticosteroid use.

Several differences in baseline characteristics were reported between compliant and non compliant patients as well as between persistent and non persistent patients. In both non compliant and non persistent groups there were significantly more patients with a prior history of diabetes (p < 0.001) or chronic renal insufficiency (p < 0.01) as well those who had previously taken estrogens, estrogen combinations or oral glucocorticosteroids (p < 0.001).

The second study was performed in the Netherlands, with data retrieved from the PHARMO Record Linkage System. A nested case control study design evaluated the association between persistent bisphosphonate use and the risk of hospitalisation for osteoporotic fractures [25]. A cohort of 14,760 bisphosphonate naive women initiated on alendronate, risedronate or etidronate was retrieved. From this cohort 541 (3.7%) cases were identified who had been hospitalised for an osteoporotic fracture after commencing treatment. The level of persistence with bisphosphonates in this group was compared to controls (n = 5,410) who had not been hospitalised for an osteoporotic fracture. One year persistent use of bisphosphonates was associated with a 26% lowered risk of fracture (OR 0.74, p < 0.05) and two years persistent use was associated with a 32% lowered risk (OR 0.68, p < 0.05). Covariates that had been adjusted for in these analyses were age, year of fracture, previous osteoporotic fracture and use of analgesics or antidepressants between the index date and fracture date.

In this study, cases were significantly older than the control population with a higher proportion being over 60 years (p < 0.0001). Cases also included fewer users of weekly bisphosphonates. A greater number had also experienced a previous fracture (p = 0.002) or had taken HRT more frequently (p = 0.045) in the one year period before the index date.

The data from only one abstract [28] has been fully reported in a peer reviewed journal [26] the others were preliminary findings [27,29-34]. The results however, endorsed the findings from the published studies, reporting an approximate 25% lower risk of fracture in patients who were compliant or persistent with bisphosphonates for at least 2 years [30,31]. The risk of hospitalisation for osteoporotic fracture was also significantly lower, [28] as was the length of hospital stay [33] and associated healthcare expenditure [32].

## Discussion

Currently available evidence on the association between compliance and persistence with bisphosphonates and fracture risk in clinical practice is limited to only two studies. However, both have reported that if bisphosphonates are taken regularly and for at least a year the risk of experiencing a fracture is reduced by approximately 25%. They have therefore started to delineate the important relationship between the level of compliance and persistence and the probability of a PMO related fracture in clinical practice. The results presented in preliminary reports examining daily and weekly bisphosphonates support the above findings, reporting that higher levels of compliance reduce the risk of fracture [27-34] with a consistently high level of compliance (MPR ≥ 90%) being required for an optimal protective effect. [30].

Studies examining groups of treatments for osteoporosis, including etidronate and conjugated estrogens [13], HRT, bisphosphonates and raloxifene [18], all osteoporosis medication [22,23] have also reported the positive relationship between higher levels of compliance with osteoporosis therapy and fracture risk. Most of the studies have used somewhat arbitrary thresholds to define compliance and persistence (e.g. 80% or 90% MPR) and in future a linear analytical model may be more useful in determining a clinically relevant relationship between fracture risk and different levels of compliance and persistence.

From a public health perspective, based on data from the USA study, an estimate of the likely benefit associated with improving compliance and persistence with bisphosphonates would be a reduction of 2,200 fractures per 100,000 over two years [24]. From the results presented in the study from the Netherlands, if the proportion of women who persist with treatment for at least a year increased by 15%, then there would be 400 fewer fractures per 100,000 per year [25].

The studies reviewed highlight that compliance and persistence levels with bisphosphonates are sub-optimal [17,18] with many discontinuing treatment soon after therapy initiation [19]. A diagnosis of osteoporosis does not appear to improve compliance with bisphosphonates [24] but women with a previous history of fracture are more persistent with treatment [18]. Clinical studies have demonstrated that a number of support activities can enhance compliance and persistence including the regular provision of patient information, repeat prescription reminder services as well as nurse counseling and support programmes [35]. However, there does not appear to be a systematic or sustained implementation of such activities in clinical practice and preliminary evidence indicates that take-up is low (~10%) [36].

The two retrospective studies of bisphosphonates will have some inherent data and analytical limitations as they were based on observational data sources. Assumptions are likely to have been made on the length of a prescription and the issuing of a prescription is only an indirect measure of medication usage and does not necessarily imply that the drug was taken in the frequency or manner expected. Therefore compliance and persistence levels could have been overestimated. In addition recording of osteoporotic fractures may not have been comprehensive, particularly if a patient had not sought medical care. Similarly, fragility fractures recorded as non traumatic may not have been validated so any count may have included some trauma fractures.

Nonetheless observational databases have been able to provide reliable estimates of patient use of medications [37] and, as the two studies examined were comparative, any bias as a result of inconsistencies in data recording would apply equally to each of the cohorts. Importantly, these resources provide access to data on real life clinical practice in large representative samples of individuals and represent a practical alternative to performing naturalistic studies.

## Conclusion

While the existing evidence is limited it does suggest that improving compliance and persistence with bisphosphonates will reduce the risk of fracture in clinical practice. In countries such as the UK where bisphosphonate regimens are the main stay of treatment [38] this could be examined using varying compliance and persistence metrics as well as a combined metric of compliance and persistence. Additionally, the protective therapeutic time window following a stop or interruption in treatment also needs be investigated.

## Competing interests

The author(s) declare that they have no competing interests.

## Authors' contributions

JA, NL, HM, MH and WC were involved in the conceptual discussion and design of the review, in the critical appraisal of the content and have given final approval to the version to be published.

## Pre-publication history

The pre-publication history for this paper can be accessed here:


